# The effect of macro- and micro-nutrient fortification of biscuits on their sensory properties and on hedonic liking of older people

**DOI:** 10.1002/jsfa.6522

**Published:** 2014-01-28

**Authors:** Roussa Tsikritzi, Paula J Moynihan, Margot A Gosney, Victoria J Allen, Lisa Methven

**Affiliations:** a Department of Food and Nutritional Sciences, University of ReadingWhiteknights, Reading, RG6 6AP, UK; b Institute for Ageing and Health, Newcastle UniversityFramlington Place, Newcastle upon Tyne, NE2 4BW; c Clinical Health Sciences, University of ReadingLondon Road, Reading, Berkshire, RG1 5AQ

**Keywords:** older people, nutrient fortification, biscuits, cookies, sensory, supplement

## Abstract

**BACKGROUND:**

Under-nutrition in older adults is widespread. Oral nutritional supplement beverages (ONS) are prescribed, yet consumption by older people is often insufficient. A variety of supplement formats may improve nutrient intake. This study developed protein and micro-nutrient fortified biscuits and evaluated their sensory attributes and liking by older people. Two micro-nutrient strategies were taken, to match typical ONS and to customise to the needs of older people.

**RESULTS:**

Oat biscuits and gluten-free biscuits developed contained over 12% protein and over 460 kcal 100 g^−1^. Two small (40 g) biscuits developed to match ONS provided approximately 40% of an ONS portion of micro-nutrients and 60% of macro-nutrients; however, the portion size was considered realistic whereas the average ONS portion (200 mL) is excessive. Biscuits developed to the needs of older adults provided, on average, 18% of the reference nutrient intake of targeted micro-nutrients. Sensory characteristics were similar between biscuits with and without micro-nutrient fortification, leading to no differences in liking. Fortified oat biscuits were less liked than commercial oat biscuits, partly attributed to flavour imparted by whey protein fortification.

**CONCLUSION:**

Macro- and micro-nutrient fortification of biscuits could provide an alternative fortified snack to help alleviate malnutrition in older adults. © 2013 Society of Chemical Industry

## INTRODUCTION

Under-nutrition is currently estimated to effect 1.3 million people over 65 years of age in the UK.^1^ Oral nutritional supplement beverages (ONS) are liquid foods that are used to improve nutritional intake in older adults and patients with a variety of health and eating problems. In a wide variety of hospital and community patients at risk of under-nutrition, the use of ONS has been shown to improve energy and nutrient intake, increase body weight and functional outcomes, reduce mortality and complications, and reduce length of stay in hospitalised patients when compared with routine clinical care.^2^ However, frail older adults with low food intakes can find it difficult to consume sufficient ONS, with reasons such as taste and volume given, to the extent that even with feeding assistance by trained staff their nutritional status is not improved.^3^ It is therefore generally accepted that offering a variety of supplements with different sensory characteristics (appearance, flavour, texture, consistency and composition) is likely to improve compliance and intake more than when only one type of supplement is used, especially for longer-term use when ‘taste fatigue’ can develop.^4^

Meal fortification can improve energy (+26%) and protein (+23%) intake in hospitalised older adults,^5^ and addition of energy-dense ingredients to regular meals is an inexpensive way to improve voluntary energy intake.^6^ It has been suggested that it is possible for older patients to achieve their nutritional needs using a combination of smaller portions of increased energy and protein density and between-meal snacks.^7^

Concerning micro-nutrients, the UK Food Standards Agency (FSA) provided guidelines in 2006 for food provision for older adults in residential care^8^ which recommended levels for five minerals (sodium, potassium, magnesium, iron and zinc) and three vitamins (riboflavin, vitamin D and folate). Bates *et al.*^9^ found iron, vitamin D, folate and vitamin C to be both low in intake and biochemically deficient in elderly institutionalised adults. Russell and Suter^10^ concluded that US recommended dietary allowances (RDAs) for vitamin D, riboflavin, vitamins B_6_ and B_12_ should be increased. These two studies strongly suggest an increased requirement for vitamin C in older people.

However, when considering fortification of foods with micro-nutrients it is important to consider that vitamins and minerals can impart tastes (such as bitter, salty, sour, sweet and metallic) and mouth-feel properties (such as astringency) to food products.^11^,^12^ It is not known whether the sensations associated with micro-nutrient fortification impact on the sensory properties of biscuits or biscuit liking in older adults. Overall, studies of taste sensitivity amongst older people show a gradual decline with age, with taste thresholds (e.g. salt and sweet) increasing;^13^ however, the extent of the decline and whether it has an impact on food selection has been debated by many authors.^14^–^16^ The loss of gustatory and olfactory acuity is highly variable between individuals.^16^ Certain medical conditions, and some drugs, may also impair the senses of taste and smell and thereby reduce appetite, leading to a reduction in food intake.^17^ However, sensory decline may be to the advantage of micro-nutrient supplementation in biscuits for older people; it may be that potentially negative tastes are below the threshold level for most older hospital patients.

The hypothesis of this study was that a protein-fortified biscuit of high energy density, supplemented with vitamins and minerals needed by older adults could be developed and would be acceptable to them. The aim was to develop a biscuit with sufficient fat and sugar levels to exhibit the functional properties of a typical short dough product, to enrich it with whey protein and to fortify it with micro-nutrients. The objectives were to (1) develop protein and micro-nutrient fortified biscuits; (2) investigate two strategies of micro-nutrient fortification by (a) matching a typical ONS beverage and (b) incorporating nutrients known to be needed by older adults at one third of the daily requirements; (3) develop a gluten-free alternative suitable of patients with coeliac disease; (4) obtain sensory profiles of the biscuits; and (5) assess consumer liking of the fortified biscuits to determine their potential as a commercial product to help meet the nutritional needs of older people.

## MATERIALS AND METHODS

### First trial materials and biscuit processing conditions

Oat biscuits with and without micro-nutrient fortification; (‘control oat biscuit’, ‘oat biscuit with ONS premix’ and ‘oat biscuit with OP premix’) and gluten-free (GF) biscuits (without micro-nutrient fortification) were prepared (Table 1). For the oat biscuits plain flour (protein 10.4%, fat 1.3%) and rolled oats were used (protein 11.0%, fat 8.1%). For gluten-free biscuits, the wheat flour was replaced by a blend of wheat-free flour including white rice flour (protein 6.5%, fat 1.0%), brown rice flour (protein 6.7%, fat 2.8%) and soya flour (protein 39%, fat 20%). For both recipes brown sugar, unsalted butter (minimum 82% butterfat), baking powder and food-grade glycerin were used. All ingredients were supermarket own-label from a local retailer (Reading, UK). Whey protein isolate (WPI) (Volac, Royston, UK) was used in order to increase the protein content of biscuits (protein 94%, fat 0.2%). Xanthan gum (X85MC, 80 mesh food grade; Chemcolloids Ltd, Congleton, UK) was added into the gluten-free flour blend.

**Table 1:** Concentration of ingredients used in biscuit formulations (g kg^−1^)

Ingredient	Control oat biscuit	Oat biscuit with ONS premix	Oat biscuit with OP premix	Gluten-free biscuit
Unsalted butter^a^	254	255	254	280
Brown sugar	201	202	201	222
Wheat flour, plain white	188	189	188	—
Oats	188	189	188	—
Rice flour, white	—	—	—	166
Rice flour, brown	—	—	—	83
Water	81	62	81	104
Whey protein isolate (WPI)	77	77	77	83
Soy flour	—	—	—	42
Baking powder	8	8	7	8
Glycerin	3	3	3	3
Xanthan gum	—	—	—	8
ONS Premix^b^	—	15	—	—
OP Premix^b^	—	—	1	—
Total	1000	1000	1000	1000

a Unsalted butter was replaced with vegetable fat (300 g) for the biscuits manufactured in trial 2 (control, ONS and OP version).

b ONS Premix = vitamin and mineral premix to match an oral nutritional supplement beverage; OP Premix = vitamin and mineral premix designed to match the needs of older people (see Table 2).

ONS, oral nutritional supplement.

In order to fortify the oat biscuits with vitamins and minerals two approaches were taken. The first approach aimed to deliver a proportion of micro-nutrients comparable to a portion of a typical ONS beverage (ONS version). The second approach (OP version) aimed to supplement biscuits at a level equivalent to a proportion of the daily reference nutrient intake (RNI) according to FSA recommendations (RNI) (OP version). The micro-nutrient premixes used are noted in Table 2 (Lycored Ltd, Aylesford, UK).

**Table 2:** Micronutrient content of premixes per 100 g of dough

	First and second trial (ONS version)		First trial (OP version)	Second trial (OP version)	
Micronutrient	1500 mg ONS premix provided:^a^	% RNI provided per 100 g dough	100 mg OP premix provided:	100 mg OP premix + 1.13 g KH_2_PO_4_ + 0.08 mg MgO provided:	% RNI provided per 100 g dough
**Minerals**					
Potassium (mg)	131	6.6	—	509	17
Calcium (mg)	221	27.6	—	—	—
Phosphorus (mg)	263	37.6	—	—	—
Magnesium mg)	62	16.5	—	50	22
Iron (mg)	4.7	33.6	4.0	4.5	48
Zinc (mg)	3.9	39	—	4.8	48
Copper (mg)	0.5	50	—	—	—
Fluoride (µg)	0.3	8.6	—	—	—
Selenium (µg)	16	29.1	—	—	—
Chromium (µg)	19	47.5	—	—	—
Iodine (µg)	29	19.3			
**Vitamins: water soluble**					
Thiamin (B_1_) (mg)	0.212	19.3	—	—	—
Riboflavin (B_2_) (mg)	0.437	31.2	0.55	0.55	39
Niacin (B_3_) (mg)	5.2	32.5	—	—	—
Pantothenic acid (B_5_) (mg)	1.7	28.3	—	—	—
Vitamin B_6_ (mg)	0.473	33.8	0.54	0.54	39
Folic acid (B_9_) (µg)	72	36	95	95	32
Vitamin B_12_ (µg)	1.0	40	—	—	—
Biotin (B_7_) (µg)	21	42	—	—	—
Vitamin C (mg)	27.0	33.8	20.0	20.0	25
**Vitamins: fat soluble**					
Vitamin A (retinol) (µg)	50	6.3	—	—	—
Vitamin D (µg)	1.8	36	4.8	4.8	96
Vitamin E (*α*-tocopherol) (mg)	2.8	23.3	—	—	—
Vitamin K (µg)	18	24	—	—	—

a The 11 minerals and 13 vitamins, at the levels provided in the 1500 mg premix, were equivalent to the vitamin and mineral composition of 200 mL of a typical ONS beverage. There were five exceptions where less was added to the biscuit dough to account for micronutrients already present in the dough ingredients: in a typical ONS beverage K 353 mg, Ca 333 mg, I 41 µg, vitamin B_1_ 0.4 mg, vitamin A 314 µg, vitamin E 3.6 mg. A typical ONS calculated as average composition of four different ONS beverages available in the UK.

ONS, oral nutritional supplement; RNI, reference nutrient intake.

Biscuit dough was mixed in three stages using a batch mixer with creaming attachment. Stage 1 involved creaming the sugar and fat (medium speed, 5 min). At stage 2, warm water and glycerin were added (low speed, 30 s). Stage 3 was the addition of all dry ingredients, which had been pre-blended into the flour (low speed, 2 min). Biscuit dough was hand-sheeted (thickness 0.5–1 cm) and cut using a 6 cm diameter round cutter. Dough pieces were placed onto solid baking sheets and baked at 190 °C in an electric rotary baking oven for 9–9.5 min. The oven temperature was monitored constantly throughout baking by using a thermocouple. Biscuits were allowed to cool, vacuum packed in three layer pouches with a polyamide barrier layer, and stored in the dark. The biscuit diameter was monitored (measuring five biscuits at a time) and the moisture content of ground biscuits was measured using an infrared drying moisture meter (MA150 with a ceramic radiator; Sartorius Mechatronics Ltd, Epsom, UK). The aim was for biscuits to be approximately 8 cm diameter and weigh between 30 and 35 g.

### Second trial materials and biscuit processing conditions

During the second trial butter was replaced by vegetable fat (300 g kg^−1^ dough) (Biscuitine; Loders Croklaan, Netherlands) upon advice from a commercial biscuit manufacturer. In addition, the OP version was further enriched with potassium, magnesium and zinc (Table 2). These minerals were not incorporated in the first trial as they were not shown to be deficient in the Bates study^18^ but their recommendation in the FSA guidelines^8^ underscores their importance; thus, they were included in the second trial. The use of dipotassium hydrogen phosphate (K_2_HPO_4_) (45% K by weight) (5.16% (w/w) addition), and magnesium oxide (MgO) (60% Mg by weight) (0.34% (w/w) addition) would provide one-third RNI of potassium and magnesium in 100 g of biscuit dough. However, due to excessive bitter and metallic tastes, these concentrations were optimised to 1.13% and 0.08% (w/w) for K_2_HPO_4_ and MgO, respectively, following tasting trials, thus resulting in 17% and 22% of RNI for potassium and magnesium respectively per 100 g.

The remaining ingredients were unchanged from the first trial. No GF biscuit was produced in this trial. In the second trial a commercial pilot plant continuous line was used. The dough was batch mixed in a three stage process as in Trial 1, but using a large batch size (15 kg compared to 2 kg). The dough was rotary deposited onto a continuous solid steel band and the biscuits baked in a continuous gas oven for 11.5 minutes. The temperature profile varied across the oven, set across three zones at 190 °C, 195 °C and 190 °C. The dough responded differently to the commercial pilot plant continuous line (Trial 2) compared to the laboratory ‘batch-process’ conditions (Trial 1) and the added water level needed to be reduced to facilitate processing. Biscuits were cooled, measured, packed and stored as in Trial 1. The aim was for biscuits to be approximately 6 cm diameter and weigh approximately 20 g.

### Nutritional profile

The nutritional profile of the biscuits was determined using the Dietplan 6 (Forestfield Software Ltd, Horsham, UK).

### Sensory analysis

Sensory profiling of biscuits was conducted by a panel of 11 trained panellists (nine female, two male, mean age 47 years) who developed a consensus vocabulary for all samples. Unlike the hedonic analysis described below, the sensory panel were not required to be over 60 years of age; they were screened for sensory acuity and trained. They did not represent the target older age group, their purpose was to provide a consistent measure of the change in product attributes as a response to ingredient change. Attribute scoring was done on 100 mm unstructured line-scales (‘not at all’ to ‘extreme’) using Compusense® software (version 5.0, release 4.8; Compusense, Ontario, Canada). Panellists were seated in individual testing booths under artificial daylight. Samples (one biscuit per person per sample) were presented in a balanced order and blinded using three-digit number codes, panellists were asked to taste at least half of the portion size. Warm water (filtered) was used as a palate cleanser and the time delay between samples (post after-effects scoring) was 30 s. For biscuits from each trial, scoring was carried out in duplicate on two separate days.

### Hedonic liking test 1

Thirty-six healthy older volunteers participated in this session (age range 62–87, mean age 71, 53% female), recruited from a university database of older adults (Berkshire, UK) who have agreed to be contacted regarding research studies. They were offered the four different types of biscuit from the first trial and asked to taste and evaluate each individually, cleansing their palate with water between samples. The participants rated their liking of each biscuit on a nine-point category scale ranging from dislike extremely to like extremely. Consumers were seated at tables in a central location. Samples were presented in a balanced order and blinded using three-digit number codes.

### Hedonic liking test 2

Twenty community dwelling elderly participants (age range age 61–78, mean age 69, 75% female) were recruited through advertisement of the study at a University of the Third Age (U3A) event in Lancashire. They tasted both the ONS version biscuit from Trial 2 and a commercial oat biscuit (Elkes oat biscuits; Fox's, Uttoxeter, UK), chosen as an in-market oat biscuit (pack declaration 27% oatflakes) product that was not fortified. The biscuits contained 481 kcal and 6.5 g protein 100 g^−1^, compared to the study biscuit at 513 kcal and 12.4 g protein 100 g^−1^. The participants rated their liking as in Trial 1.

In both liking tests the consumers were naïve to the type of biscuits being sampled. Their participant information sheet informed them that the study concerned ‘Improving the palatability of nutrition feeds & foods in order to reduce malnutrition in adults’. The participants were not a diverse socio-economic group; they were recruited from groups interested in research. The project was given approval to proceed by the University of Reading Research Ethics Committee (study number 08/30).

### Statistical analysis

Statistical analysis of data was performed with Senpaq (SenPaq, v4.2; Qi Statistics Ltd, Reading, UK) and XLStat (version 2009; Addinsoft, Paris, France). Profiling data was normally distributed and analysed by two-way ANOVA (treatments: sample and assessor) and Fisher's LSD test was used for multiple comparisons. The Kruskal–Wallis test was used to analyse differences in liking scores from the four samples from Trial 1. The Wilcoxon signed rank test was used in the second consumer trial where only two samples were presented.

## RESULTS

Concerning the physical characteristics of the biscuits; the control, ONS, OP and GF biscuits in Trial 1 had mean diameters of 8.3 ± 0.3, 7.2 ± 0.2, 9.0 ± 0.4 and 8.5 ± 0.1 cm, respectively, and weighed 33 ± 1 g. The moisture content of all biscuit types after 4 weeks storage was less than 5%. It was considered that the portion size of the Trial 1 biscuits were too large (as highlighted by comments from the older consumers) and, therefore, Trial 2 biscuits (control, ONS and OP versions) were of smaller diameter, all at 6 ± 0.1 cm, and weighed 20 ± 0.8 g. The moisture content post baking was 2.3–2.6%.

A summary of the nutritional profile of the biscuits is presented in Table 3. Protein content of the biscuits varied from 12.3 to 12.7 g 100 g^−1^ baked weight and energy content between 462 and 522 kcal 100 g^−1^. Biscuits from the second trial contained higher energy levels (ca. 520 kcal 100 g^−1^), resulting from the change from butter to vegetable fat which increased the fat content up to 33 g 100 g^−1^ (rather than 24 g in Trial 1).

**Table 3:** Nutrient profile of biscuits (first and second trials)

	First trial				Second trial		
Parameter	Control oat biscuits	ONS premix oat biscuits	OP premix oat biscuits	Gluten-free biscuits	Control oat biscuits	ONS premix oat biscuits	OP premix oat biscuits
Energy (kcal)	462	464	462	474	522	513	517
Protein (g)	12.4	12.4	12.4	12.3	12.7	12.4	12.4
Carbohydrates (g)	50.3	50.5	50.2	48.9	48	47.6	47.3
of which sugars (g)	23.1	23.2	23.1	25.4	22.2	22.0	21.8
Fat (g)	24.7	24.8	24.7	26.5	32.6	31.8	32.4
of which saturated (g)	15.4	15.5	15.4	16.8	22.4	21.8	22.3
Fibre (g)	2.7	2.7	2.7	1.4	2.6	2.6	2.5
Sodium (mg)	128	128	115	132	118	117	116
Potassium (mg)	79	223	79	195	72	207	596
Calcium (mg)	90	332	89	82	88	315	86
Phosphorus (mg)	116	406	107	159	109	380	107
Magnesium (mg)	12	80	12	26	12	76	65
Iron (mg)	0.87	6.02	5.27	1.35	0.85	5.71	5.33
Zinc (mg)	0.59	4.85	0.59	0.69	0.62	4.65	5.40
Copper (mg)	0.47	1.06	0.47	0.66	0.50	1.01	0.49
Selenium (µg)	0.5	0.5	0.5	0.5	0.5	17.5	0.5
Iodine (µg)	2.1	18.5	2.1	—	2.0	32.9	1.9
Thiamin (B_1_) (mg)	0.06	0.29	0.06	0.06	0.05	0.28	0.05
Riboflavin (B_2_) (mg)	0.02	0.5	0.62	0.04	0.02	0.48	0.57
Niacin (B_3_) (mg)	0.33	6.07	0.33	0.63	0.32	5.86	0.31
Pantothenic acid (B_5_) (mg)	0.05	1.88	0.05	0.06	0.05	1.86	0.04
Vitamin B_6_ (mg)	0.02	0.55	0.62	0.06	0.02	0.53	0.56
Folic acid (B_9_) (µg)	2	82	107	8	2	79	97
Vitamin B_12_ (cobalamin) (µg)	—	0.8	—	—	—	1.1	—
Biotin (B_7_) (µg)	0.2	23	0.2	—	0.2	22.6	0.2
Vitamin C (mg)	—	30	22	—	—	29	20
Vitamin A (retinol) (µg)	—	—	—	—	—	53	—
Vitamin D (µg)	—	1.95	5.27	—	—	1.92	4.8
Vitamin E (*α*-tocopherol) (mg)	0.06	3.14	0.06	0.07	1.23	4.19	1.23

Results are given as per 100 g biscuit weight (baked).

ONS, oral nutritional supplement.

### Sensory profile results

Concerning the sensory profile, a consensus vocabulary used to describe the first trial biscuits contained 27 attributes; however, only nine attributes were found to be significantly different between the biscuits (Table 4). There was no significant difference between the control and the ONS version of the oat biscuits. The oat biscuits were all scored equally oaty and with ‘bits’. The control biscuits had more white flakes than the OP version but significantly lower doughy and rancid fat flavour and they had a less cracked appearance. Compared to the oat biscuits, the GF biscuits had higher rancid fat flavour, more cracked appearance and caused the highest greasy lips after-effect, which may be explained by the use of non-defatted soy flour. The soy flour contained 20% fat, in contrast with the plain flour which only had 1.3%. As expected, given that they did not contain oats and white flour, GF biscuits had significantly fewer white flakes and particles (‘bits’) and were lower in doughy odour and oaty flavour than all oat biscuits. Trial 2 biscuits shared a similar sensory profile to the Trial 1 oat biscuits. Thirty-two attributes were defined at the vocabulary session and on scoring 14 were significantly different between products. The ONS biscuit was found to be more unevenly baked, had a more savoury odour, and a greater burnt sugar flavour compared to the control but was less sweet in after-taste (Table 4). Both ONS and OP biscuit versions appeared less cracked and more dense, with greater burnt sugar flavour and less sweet after-taste compared to the control. As in Trial 1, the OP version differentiated from the control more than the ONS version and, therefore, only the ONS biscuit version from Trial 2 was taken forward to the second consumer trial. The OP version had a more dense appearance and was less cracked than the control; unlike in Trial 1 where the OP version was more cracked. In this trial the OP version had a sweeter smell, yet tasted less sweet and more bitter than the control. The ONS version was also more bitter than the control, but not significantly. The milk powder and oaty flavours were less intense in the OP version while the burnt sugar flavour for both OP and ONS versions was greater than in the control biscuit; not an effect found in Trial 1. The OP biscuit caused a rougher and more dense mouth-feel as well as less sweet after-taste.

**Table 4:** Mean scores of sensory attributes found to discriminate biscuit samples (0–100 scale)

Modality	Attribute	Control biscuits	ONS premix oat biscuits	OP premix oat biscuits	Gluten-free biscuits
First trial biscuits*					
Appearance	White flakes	26.1^a^	20.1^ab^	15.3^b^	2.5^c^
	Cracked	34.9^b^	41.7^b^	61.6^a^	38.8^b^
Odour	Doughy	20.0^b^	20.9^ab^	28.2^a^	17.1^b^
	Rancid fat	18.7^c^	23.5^bc^	27.0^ab^	31.5^a^
Flavour	oaty	35.2^a^	36.9^a^	34.8^a^	10.6^b^
Mouth-feel	Rough	36.4^ab^	38.4^ab^	44.2^a^	30.5^b^
	Dense	50.8^ab^	50.4^b^	59.8^a^	47.0^b^
	Bits	34.8^a^	33.5^a^	34.4^a^	14.6^b^
After-effects	Greasy lips	19.3^b^	24.0^ab^	25.5^ab^	32.1^a^
Second trial biscuits*					
Appearance	Contrast top to bottom	43.0^b^	50.4^a^	47.7^ab^	—
	Dense	46.8^b^	53.6^a^	53.8^a^	—
	Cracked	54.8^a^	44.6^b^	34.1^c^	—
Smell	Sweet	31.3^b^	29.7^b^	41.6^a^	—
	Savoury	29.9^b^	41.6^a^	29.7^b^	—
Taste	Sweet	49.9^a^	48.3^a^	42.4^b^	—
	Bitter	20.6^b^	26.6^ab^	29.6^a^	—
Flavour	Milk powder	19.1^a^	16.9^ab^	11.1^b^	—
	Oaty	38.2^a^	37.6^ab^	32.1^b^	—
	Burnt sugar	28.6^b^	41.9^a^	44.7^a^	—
Mouthfeel	Rough	40.7^b^	44.7^ab^	46.9^a^	—
	Dense	41.3^b^	42.2^ab^	50.1^a^	—
After-effects	Greasy lips	24.5^ab^	22.0^b^	26.2^a^	—
	Sweet (after-taste)	54.6^a^	44.3^b^	39.2^b^	—

a,b,cMean values with the same superscript letter in same row are not significantly different at *P* < 0.05.

* Attributes rated which were not significantly different between samples were:

In trial 1: appearance (dry, unevenly baked, dense), odour (sweet, savoury, fish oil), taste (sweet, sour, salty), flavour (dairy, flour, chemical, burnt sugar), mouth-feel (dry, crumbly, adhesive), after-effects (teeth coating, residue)

In trial 2: appearance (dry, crumbly, white flakes, dark bits), odour (oily, rancid fat), taste (sour, salty, metallic), flavour (flour, caramel), mouth-feel (dry, crumbly, adhesive), after-effects (teeth coating, bitty residue, sugar crystals, mouth-drying)

ONS, oral nutritional supplement.

### Hedonic liking results

**Figure 1:**
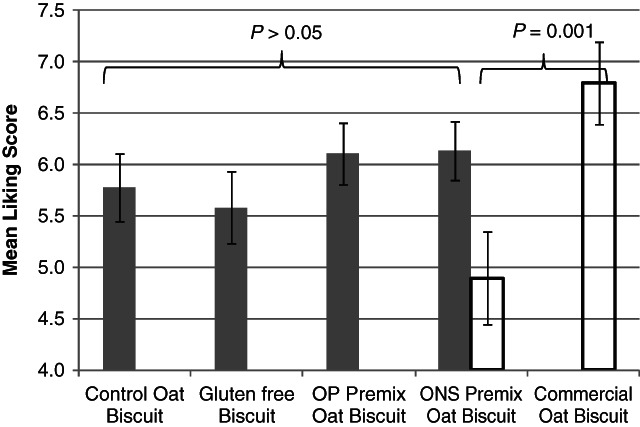
Mean hedonic liking results for biscuits by older volunteers, where Trial 1 biscuits were tested by *n* = 36 consumers and Trial 2 ONS version was tested against a commercial oat biscuit by *n* = 20 consumers. Error bars represent standard error of the mean. The mean liking score was on a scale of 1 to 9, where 1 = dislike extremely; 5 = neither like nor dislike; and 9 = like extremely. White bars, Trial 1; dark grey bars, Trial 2. ONS, oral nutritional supplement.

Figure 1 displays the mean liking scores given by older consumers for Trial 1 biscuits and for the ONS version of the oat biscuit from Trial 2 against a commercial oat biscuit. From Trial 1, there were no statistically significant differences in liking between the different types of biscuit. It therefore appears that the slight differences in sensory characteristics found between the micro-nutrient enhanced biscuits and the control, and between the gluten-free biscuit and the oat control biscuit (Table 4), did not lead to differences in mean liking scores. The higher levels of rancid fat odour in some of the biscuits may not have been detected by many of the older consumers as it did not affect mean liking scores.

The second consumer trial compared the ONS version of the oat biscuit from Trial 2 with a commercial oat biscuit. The commercial biscuit was significantly more liked than the trial biscuit (mean liking score ± standard deviation of 6.8 ± 1.3 compared to 4.9 ± 2.3) (*P* = 0.001).

## DISCUSSION

The biscuits produced in this study were of the short dough type, typically made from soft and weak wheat flours and characterised by a formula high in sugar and fat. The high quantities of sugar and fat in the dough allow plasticity and cohesiveness without the formation of an elastic gluten network.^19^ Biscuits usually show a substantial increase in diameter during baking that is attributed to the gluten which forms a collapsible film.^20^ The GF biscuit increased similarly in size post baking due to xanthan gum which can mimics the visco-elastic properties of gluten.^21^

Short dough biscuits with fat contents over 20% are the most widespread type of biscuits on the market.^22^ Protein content is usually low as higher protein contents lead to harder biscuits. In a previous paper, the protein content of a standard short dough biscuit was 4.8% (w/w) and the energy content was 448 kcal 100 g^−1^.^19^ Higher fat, protein and energy levels are found in products such as commercial shortcake biscuits, at 28.4 g 100 g^−1^, 6.3 g 100 g^−1^ and 520 kcal 100 g^−1^, respectively.

In Trial 1, all types of biscuits were higher in protein than standard biscuits (12.3–12.4% w/w) and contained high amounts of fat and carbohydrates (Table 3). Energy content varied between 462 kcal 100 g^−1^ for the oat biscuits and 474 kcal 100 g^−1^ for the GF biscuits. A portion size of the biscuits was considered to be 65 g (two biscuits) equating to 306–317 kcal per portion. Typical ONS beverages have energy contents of 138 kcal 100 mL^−1^ equating to 275 kcal per portion. The energy content of the Trial 1 biscuits was, therefore, slightly higher than the energy content of a typical ONS beverage; however, comments from the older volunteers in the liking trials indicated that the portion size was too large, so this was modified to 40 g in the second trial. From the second trial formulations, a portion size of two biscuits (40 g) provided 209 kcal (Table 5). A typical commercial short dough biscuit weighs 14–15 g, and they are often sold as individual packs of three biscuits. Through provision of approximately 200 kcal in a realistic biscuit portion size, we believe that compliance for product would be high and, therefore, this energy intake would be achieved. In comparison, poor compliance with ONS has been demonstrated previously with 63% of ONS being wasted when monitored on an elderly care ward.^23^

**Table 5:** Proportion of daily reference nutrient intake^a^ for older people supplied by one portion of biscuits (second trial), and compared to a typical oral nutritional supplement (ONS) beverage

	Nutrients per portion (40 g biscuits, 200 mL beverage)					% of Daily RNI^a^ within a portion			
Nutrient	RNI^a^	Control oat cookie	ONS cookie	OP cookie	Typical^d^ ONS beverage	Control oat cookie	ONS cookie	OP cookie	Typical^d^ ONS beverage (200 mL)
**Micronutrients**									
Vitamin D (µg)	10	ND	0.77	1.92	2.0	ND	8	19	20
Riboflavin (B_2_) (mg)	1.2	0.01	0.19	0.23	0.5	0.7	16	19	41
Vitamin B_6_ (mg)	NR (1.3)^b^	0.01	0.21	0.22	0.5	0.6	16	17	41
Folate (B_9_) (µg)	200	1	32	39	77.3	0.4	16	19	39
Vitamin C (mg)	NR (40)^b^	ND	12	8.00	26.9	ND	29	20	67
Potassium (mg)	3500^c^	29	83	238	353	0.8	2	7	10
Magnesium (mg)	300	5	30	26	62	1.6	10	9	21
Iron (mg)	9	0.34	2.28	2.13	4.7	3.8	25	24	52
Zinc (mg)	9.5	0.25	1.86	2.16	3.9	2.6	20	23	41
**Macronutrients**									
Energy (kcal)	1955	209	205	207	275	11	10	11	14
Protein (g)	50	5.1	5.0	5.0	13.6	10	10	10	27
Fat (g)	<74.5	13.0	12.7	13.0	9.2	18	17	17	12
Saturated fatty acids (g)	<23.5	9.0	8.7	8.9	1.2	38	37	38	5

a Food Standards Agency, UK (2006) guidelines for nutrients for food provided to older people in residential care.

b NR, no recommendation specified; but highlighted as low intake and/or deficient in older adults.^10^,^18^

c Except in cases of renal disease where the daily RNI < 274 mg.

d Typical ONS calculated as the average composition of four different ONS beverages available in the UK.

ND, not detected; RNI, reference nutrient intake.

OP premix: a vitamin and mineral premix designed to match the needs of older people (see Table 2).

The oat biscuits contained 7.7 g of WPI (95% protein), 18.8 g soft wheat flour (9% protein) and 18.8 g oats (17% protein) per 100 g dough. Therefore the 11.7–12.7% protein in the final baked products comprised 59.5% whey protein, 14.1% wheat protein and 26.4% oat protein. Whey protein has been found to stimulate muscle protein synthesis better than other protein sources.^24^ The percentage of energy from protein was 10% for the Trial 1 biscuits, whereas in a typical commercial shortcake biscuit it is 4.8%.

The fat content of the study biscuits was 24–32 g 100 g^−1^, of which saturated fats were 13.5–22.4 g 100 g^−1^. This was above the FSA guidelines for older people (maximum total % energy from fat 35%, from saturated fat 11%); however, the guidelines were not written specifically for malnourished older adults where a higher intake of fat and sugar in the short term to boost energy intake is often acceptable. In addition, the fat levels in the study biscuits were in line with a typical short dough shortcake biscuit where a commercial declaration per 100 g is 28.4 g fat and 13.6 g saturated fat.

This study used two approaches to micro-nutrient fortification. In both trials, the ONS version of the biscuit was fortified with 11 minerals and 13 vitamins compared with the control. The ONS version had approximately double the concentration of vitamins and minerals per 100 g when compared with 100 mL of a typical (average) ONS beverage. However, the small portion size (40 g) meant that one portion of ONS biscuits provided approximately 40% of an ONS 200 mL portion of vitamins and minerals (Table 5); although the portion size was thought to be more likely to be consumed. The OP version during the first trial had fortified levels of iron (Fe) and vitamins riboflavin (B_2_), B_6_, folic acid (B_9_), vitamin D and, in the second trial, potassium, (K), magnesium (Mg) and zinc (Zn). These were justified based on the FSA guidelines^8^ as well as on the studies by Bates *et al.*^18^ and Russell and Suter^10^ (outlined in Table 5). The aim of the OP biscuit version was to provide one-third of the daily requirements of micro-nutrients determined to be most necessary to fortify products for older people, assuming three servings of fortified biscuits would be provided to under-nourished patients per day. Table 5 demonstrates that both ONS and OP versions of the oat biscuit were substantially higher in these required micro-nutrients than the control. However, as the acceptable portion size was small the proportion of RNI achieved was approximately one-fifth for the vitamins (17–20%) and one quarter for Fe and Zn (24%, 23%) rather than one-third. Levels of K and Mg were lower as initial screening trials found that higher levels led to an unacceptable taste. Therefore, the proportion of the daily RNI achieved for K and Mg were 7% and 9%, respectively (OP version).

The dairy flavour developed in all biscuits was attributed to the addition of WPI. The Trial 1 biscuits had dairy flavour mean values ranging from 25 to 31 out of 100 (data not shown). In Trial 2 the dairy note (described as milk powder flavour) was higher in the control than in the micro-nutrient fortified versions.

Addition of vitamins and minerals caused some differences in the sensory characteristics of the micro-nutrient fortified biscuits; however, a consistent trend was not found across the two trials. An increase in bitter taste is likely to result from micro-nutrient fortification; this was found to be significant for the OP version of the biscuits in Trial 2 where potassium and magnesium enrichment was highest.

There were two main differences between the Trial 1 and Trial 2 biscuits; in the second trial vegetable fat was used rather than butter (leading to a higher level of total fat), and the biscuits were manufactured by a continuous pilot plant process rather than by the batch laboratory process. The change in process conditions might have led to further Maillard reaction which can be promoted by minerals and this may be responsible for the higher levels of burnt sugar flavour in the ONS and OP biscuits from the second trial. Cracked appearance was found to increase with mineral inclusion in Trial 1 but decreased with minerals in Trial 2; this may be due to batch to batch variation but may be dependent on aqueous to lipid phase ratio. In Trial 1 (lower fat than Trial 2) the minerals may have affected the rheology of the aqueous phase, changing dough structure and subsequent collapse sufficiently to increase surface cracking.

The addition of metal ions, such as iron, may have been responsible for the higher rancid odour noted in the Trial 1. Iron is a pro-oxidant and may also catalyse the oxidation of vitamins A and C.^25^–^27^ Addition of retinyl acetate and retinyl palmitate as vitamin A salts are effective at increasing baking and storage stability and addition of vitamin A salts have previously been found not to alter the colour and flavour of biscuits.^28^ However, taste, texture, appearance and overall acceptability may decrease as a function of storage.^28^,^29^ The ONS version of the biscuits contained vitamin A and antioxidant in the form of *α*-tocopherol (vitamin E) which the OP version did not. This might have reduced lipid oxidation compared to the OP version, although they were not significantly different from each other. Although differences in rancid fat flavour were not found in Trial 2 (vegetable fat used in place of butter), a shelf life study of the fortified biscuits should be carried out before commercialisation.

Overall liking scores from the older consumers (*n* = 36, test 1; *n* = 20, test 2) for the fortified cookies ranged from 4.9 to 6.1 on a nine-point scale. Although significant differences were not found between the biscuits in test 1 with this number of older participants, the nine-point hedonic scale has previously been used successfully with an older-age cohort of this size to discriminate between liking of products^30^ and indeed the scale did lead to significant differences in test 2. The pilot plant product was out-performed by the commercial product in test 2. This might imply that the older consumers liked the ONS version of the oat biscuit less because they could detect negative attributes such as bitter and metallic taste or burnt sugar flavour, or that they did not like the dairy (milk powder) flavour in the trial biscuit that would not have been in the commercial product where no milk powders were added. It is not clear why the consumer group tasting the biscuits from the first trial scored the ONS version of the biscuit substantially higher in mean liking than a different consumer group scoring the same product formulation from the second trial (mean 6.1 compared to 4.9). It may be a difference between the two separate consumer groups, or half of the second group may have been influenced by the commercial cookie which they tasted first, or because the Trial 2 pilot plant produced ONS version was sensorially more different from the control than the batch produced biscuits in Trial 1 (Table 4). However, in the first trial all biscuits contained the WPI and there were no differences in mean liking between the micro-nutrient fortified and control biscuits. Therefore, it is thought less likely that the micro-nutrient fortification was the main cause of lower liking score for the ONS version of the biscuit compared to the commercial biscuit in the second hedonic test. Although a significant difference in mean liking such as that found in test 2 (1.9 on a nine-point scale) might be expected to lead to a significant difference in consumption, this subject is not covered widely in the literature. Most studies with older cohorts have either investigated consumption or liking, but have not quantified whether a significant difference in liking leads to a significant difference in consumption.

In a study conducted by Ranhotra and co-workers, high protein (15.7 g 100 g^−1^) biscuits were developed. Product acceptability ratings were only carried out by eight assessors, resulting in mean scores of approximately 5 out of 9,^31^ similar to the liking scores recorded in the present study. In the Ranhotra study protein enrichment was achieved by addition of defatted soy flour and peanut butter. However, in the present study whey protein was incorporated as it is known to lead to more effective stimulation of muscle protein synthesis. A more recent study by Boobier *et al.*^19^ considered commercially manufactured biscuits enhanced with vitamins, but with lower protein content (6% w/w). A more limited range of micro-nutrients were used to fortify the biscuits than in the current study; however, the levels added were higher with one biscuit providing 92, 100, 174 and 135% of the UK RNI for vitamins C, B_6_, folic acid and B_12_, respectively. A consumer test (*n* = 25, student age group) found no difference in liking between fortified and non-fortified samples (mean liking scores 7.4 and 7.6, respectively, on a nine-point scale). It is, therefore, likely that the levels of these vitamins could be further increased in the present study without detriment to product acceptability. A current commercial breakfast oat biscuit (Belvita; Kraft Foods, Uxbridge, UK) has incorporated a range of cereals plus skimmed milk powder leading to a protein content of 8 g 100 g^−1^ (rather than >12 g 100 g^−1^ in the present study) and fortified the product with vitamins B_1_, B_3_, iron and magnesium at similar levels to biscuits in the present study.

## CONCLUSION

This study successfully developed protein-fortified oat biscuits and gluten-free biscuits, with protein contents varying from 12.3% to 12.7% in comparison to typical short dough biscuits at 6–7% protein. Micro-nutrients were incorporated into the oat biscuits by two approaches. The first was to add a wide range of vitamins and minerals to replicate the approach taken by typical ONS beverages. The second approach was to add micro-nutrients that were customised to the needs of older adults in hospital (riboflavin, vitamin B_6_, folic acid, vitamin C, vitamin D, iron, potassium, magnesium and zinc). The micro-nutrient fortification led to relatively minor differences in sensory attributes and older consumers liked equally the protein-fortified biscuits with and without micro-nutrient fortification, although the findings should be confirmed with a larger consumer group of older adults. However, a comparison of protein-fortified and micro-nutrient-enriched oat biscuits against a commercial oat biscuit found the trial biscuits to be significantly less liked. Further optimisation of the protein- and micro-nutrient-fortified biscuit is therefore required in order to commercialise this concept, and shelf life testing must be carried out. Overall, however, the use of whey protein to increase product protein levels, and fortification with micro-nutrients customised to the needs of older people can lead to acceptable products. This is recommended as a way forward to diversify the nutritionally supplemented products available for older adults at risk of malnutrition.
